# Novel role for endogenous mitochondrial formylated peptide-driven formyl peptide receptor 1 signalling in acute respiratory distress syndrome

**DOI:** 10.1136/thoraxjnl-2017-210030

**Published:** 2017-05-03

**Authors:** David A Dorward, Christopher D Lucas, Mary K Doherty, Gavin B Chapman, Emma J Scholefield, Andrew Conway Morris, Jennifer M Felton, Tiina Kipari, Duncan C Humphries, Calum T Robb, A John Simpson, Phillip D Whitfield, Christopher Haslett, Kevin Dhaliwal, Adriano G Rossi

**Affiliations:** 1 The MRC Centre for Inflammation Research, Queen's Medical Research Institute, University of Edinburgh, Edinburgh, UK; 2 Department of Diabetes and Cardiovascular Science, Division of Health Research, University of the Highlands and Islands, Inverness, UK; 3 University Division of Anaesthesia, Addenbrooke's Hospital, Cambridge, UK; 4 Institute of Cellular Medicine, Newcastle University, Newcastle upon Tyne, UK

**Keywords:** Neutrophil Biology, ARDS, Innate Immunity

## Abstract

**Background:**

Acute respiratory distress syndrome (ARDS) is an often fatal neutrophil-dominant lung disease. Although influenced by multiple proinflammatory mediators, identification of suitable therapeutic candidates remains elusive. We aimed to delineate the presence of mitochondrial formylated peptides in ARDS and characterise the functional importance of formyl peptide receptor 1 (FPR1) signalling in sterile lung inflammation.

**Methods:**

Mitochondrial formylated peptides were identified in bronchoalveolar lavage fluid (BALF) and serum of patients with ARDS by liquid chromatography–tandem mass spectrometry. In vitro, human neutrophils were stimulated with mitochondrial formylated peptides and their effects assessed by flow cytometry and chemotaxis assay. Mouse lung injury was induced by mitochondrial formylated peptides or hydrochloric acid. Bone marrow chimeras determined the contribution of myeloid and parenchymal FPR1 to sterile lung inflammation.

**Results:**

Mitochondrial formylated peptides were elevated in BALF and serum from patients with ARDS. These peptides drove neutrophil activation and chemotaxis through FPR1-dependent mechanisms in vitro and in vivo. In mouse lung injury, inflammation was attenuated in Fpr1−/− mice, effects recapitulated by a pharmacological FPR1 antagonist even when administered after the onset of injury. FPR1 expression was present in alveolar epithelium and chimeric mice demonstrated that both myeloid and parenchymal FPR1 contributed to lung inflammation.

**Conclusions:**

We provide the first definitive evidence of mitochondrial formylated peptides in human disease and demonstrate them to be elevated in ARDS and important in a mouse model of lung injury. This work reveals mitochondrial formylated peptide FPR1 signalling as a key driver of sterile acute lung injury and a potential therapeutic target in ARDS.

**Key messages**

**What is the key question?**

- Are mitochondrial formylated peptides present in acute respiratory distress syndrome (ARDS) and does their signalling via formyl peptide receptor 1 (FPR1) propagate sterile lung inflammation?

**What is the bottom line?**

- For the first time, we have demonstrated that mitochondrial formylated peptides are present and elevated in ARDS and that they exert effects through FPR1 to drive sterile lung inflammation.

**Why read on?**

- Elevated levels of mitochondrial formylated peptides in ARDS and their signalling via FPR1 to immune and lung parenchymal cells drives acute lung injury, thereby highlighting a potential novel therapeutic target.

## Introduction

Acute respiratory distress syndrome (ARDS) is the often fatal common pathway of a broad range of sterile and infective aetiologies. These include direct insults to the lung parenchyma such as bacterial or viral pneumonia and aspiration of gastric contents, and distal organ injury including non-pulmonary sepsis and major trauma.^1^ Despite significant improvements in ventilation strategies and fluid management there remain no effective pharmacological therapies in routine clinical practice.^2^

As a neutrophil-dominant disorder, ARDS is characterised by a dysregulated proinflammatory environment, epithelial cell dysfunction and death, pulmonary oedema, hyaline membrane formation and hypoxia. Neutrophil migration into sites of inflammation is a highly regulated and complex process that is orchestrated by a variety of chemotactic factors and receptors with increases in neutrophil number in ARDS associated with poorer outcome.^3^

In addition to key, well described cytokines and chemokines in the pathogenesis of ARDS,^4^ understanding of the importance and role of damage-associated molecular patterns (DAMPs) is evolving. These include high-mobility group box 1 (HMGB1), fibronectin and several heat shock proteins,^5^ which serve to drive a variety of pathogenic mechanisms including increased cytokine expression, alveolar leak and neutrophil migration. Targeting their cognate receptors has therefore been proposed as a potential therapeutic approach.^5^ ^6^

One group that remains unexplored in this context are mitochondrial-derived DAMPs, namely CpG-rich mitochondrial DNA (mtDNA) and formylated peptides, which are released during cell death. Mitochondrial formylated peptides are of particular interest as much is known about their counterparts; namely bacterial formylated peptides which bind to the G protein-coupled receptor formyl peptide receptor 1 (FPR1). This interaction drives neutrophil chemotaxis and stimulates a variety of antimicrobial responses, including degranulation, reactive oxygen species production and cytokine release. FPR1 deficiency results in increased bacterial burden and mortality in models of systemic *Listeria monocytogenes* infection but, despite this, the influence of FPR1 in pulmonary models of infection is unclear.^7–10^ Due to proposed evolutionary symbiosis mitochondrial-synthesised peptides within eukaryotic cells, similar to bacterial peptides, contain a formylated N-terminus and bind FPR1 with great affinity.^11^

FPR1 is potentially a key receptor within the acute inflammatory process with the capacity to sense and respond to unique bacterial and host-derived factors. It is one member of the formyl peptide receptor (FPR) family which in humans constitutes FPR1, FPR2/ALX (lipoxin receptor) and FPR3. These are well conserved G protein-coupled receptors that have pluripotent and diverse roles in the initiation and resolution of inflammation.^12^ While FPR1 has relatively specific binding to only formylated peptides, Annexin A1 and Cathepsin G, FPR2 is a highly promiscuous receptor which can bind a variety of lipids, peptides and proteins to exert ligand-dependent pro-inflammatory or pro-resolution/anti-inflammatory effects.^13^ Therefore, we have focused on the dominant pro-inflammatory formyl peptide receptor, FPR1; future work on FPR2 would be of interest especially when investigating the resolution phases of inflammation. The role of FPR3, however, is less clear and likely plays only a subtle role in inflammation, although this still has to be fully elucidated.

Recent work has demonstrated that mitochondrial DAMPs released following cell death play an important role in the context of systemic sterile inflammation and are pivotal in neutrophil migration in liver injury.^14^ ^15^ Indeed, mtDNA is elevated in a variety of disease contexts, including systemic inflammatory response syndrome, sepsis and acute acetaminophen overdose.^14^ ^16–19^ Despite these observations characterisation of the role of mitochondrial formylated peptides and FPR1 in sterile inflammation remains limited, particularly within the lung. No definitive evidence is currently available for the presence and pathogenic role of mitochondrial formylated peptides in pulmonary inflammation. Furthermore, while FPR1 expression and function have characteristically been associated with neutrophil expression, a role for FPR1 in lung parenchymal cell function in vivo is unknown.

In this study, we therefore developed a mass spectrometry based method to determine the presence of mitochondrial formylated peptides in ARDS. Subsequently, we investigated the contribution of mitochondrial formylated peptides and FPR1 signalling in the pathogenesis of sterile lung inflammation in mice and the influence of FPR1 expression in myeloid and parenchymal cells in this context.

## Materials and methods

### Clinical samples

Bronchoalveolar lavage fluid (BALF) and serum samples from ventilated patients with ARDS in an intensive care setting and healthy volunteers were collected as part of a previously reported study.^20^ Clinical sample collection was approved by the Lothian Research Ethics Committee (LREC/2002/8/19, 06/S1101/50). Further details are provided within the online supplementary methods.

10.1136/thoraxjnl-2017-210030.supp1 supplementary data

### Analysis of N-formylated mitochondrial peptides

Samples (mitochondria (MTD), BALF and serum) were analysed by liquid chromatography–tandem mass spectrometry (LC-MS/MS) with N-formylated peptides identified based on their accurate mass, retention times and characteristic fragmentation patterns compared with custom synthesised standards (Peptide Protein Research Ltd, Fareham, UK).

### Neutrophil isolation and surface marker expression

Peripheral blood neutrophils were isolated from healthy human volunteers (Lothian Research Ethics Committee (#08/S1103/38)) with cell purity routinely ≥95%.^21^ Cells were pretreated with the FPR1 antagonist cyclosporin H (CsH) (2.5 μM, Enzo Scientific, Exeter, UK) then stimulated with 100 nM fMIT (fMMYALF; GenScript, Hong Kong), isolated MTD or vehicle control for 30 min (37°C). Neutrophils were then incubated with antibodies to CD11b/CD18/CD62L (all BioLegend, London, UK) prior to analysis by flow cytometry (BD FACS Canto, BD Biosciences, Oxford, UK). Neutrophil chemotaxis and western blotting are described in the supplemental methods.

### Mouse models of lung inflammation

Female, 8–10 week old Fpr1−/− mice (C57/Bl6J background) and wild type (WT) C57/Bl6 (Charles River or appropriate litter mate controls) were used for all experiments and housed in pathogen-free conditions. Animal work was carried out in accordance with the UK Home Office under the Animals (Scientific Procedures) Act 1986 under UK Home Office-approved project licences 60/4434 and 60/4531.

Following anaesthesia and direct, visualised intubation of the trachea,^22^ 50 μL intratracheal HCl (pH2.0, Sigma), fMIT (0.25 mg/kg) or appropriate vehicle control was instilled. In acid injury experiments a subsequent 200 μL bolus of intraperitoneal 0.9% saline was given and mice were kept in a humidified oxygen chamber until recovered from anaesthetic. For pharmacological experiments intraperitoneal CsH (5 mg/kg) or vehicle control (ethanol) was delivered either 30 min prior to intratracheal acid and again at 12 hours or only 12 hours after injury. All animals were culled 24 hours after injury and tissues processed.

### Epithelial FPR1 expression

To determine FPR1 expression WT mouse lungs were harvested and single cell digest performed.^22^ Cells were incubated with antibodies to CD324 and CD326-PE with DAPI–/CD324+/CD326– cells collected from each mouse by flow sorting (BD FACSAria II SORP, BD Biosciences), RNA extracted and RT-PCR performed. Immunohistochemical staining of formalin fixed, paraffin embedded sections of naïve WT and Fpr1−/− mouse lungs were stained with antibodies to FPR1 (Biorbyt, Cambridge, UK).

### Statistics

Flow cytometry analyses were conducted using FlowJo (Tree Star, Ashland, USA) with statistical analyses performed using GraphPad Prism (La Jolla, California, USA). Data are presented as mean±SEM and were analysed by student's t-test, Mann–Whitney test, one-way ANOVA, Kruskal–Wallis test or two-way ANOVA as appropriate. Detailed methodologies for all experiments are provided in online supplementary data.

## Results

### Mitochondrial N-formylated peptides are elevated in patients with ARDS

LC-MS/MS was used to identify the previously described formylated N-terminal hexapeptides of the 13 mitochondrial encoded proteins.^11^ Initial studies focused on mitochondria isolated from HepG2 cells. A full screen for free mitochondrial peptides revealed the presence of N-formylated termini of NADH-ubiquinone oxidoreductase chain 2 (NADH2; fMNPLAQ) and NADH-ubiquinone oxidoreductase chain 4 L (NADH4L; fMPLIYM).

**Figure 1:**
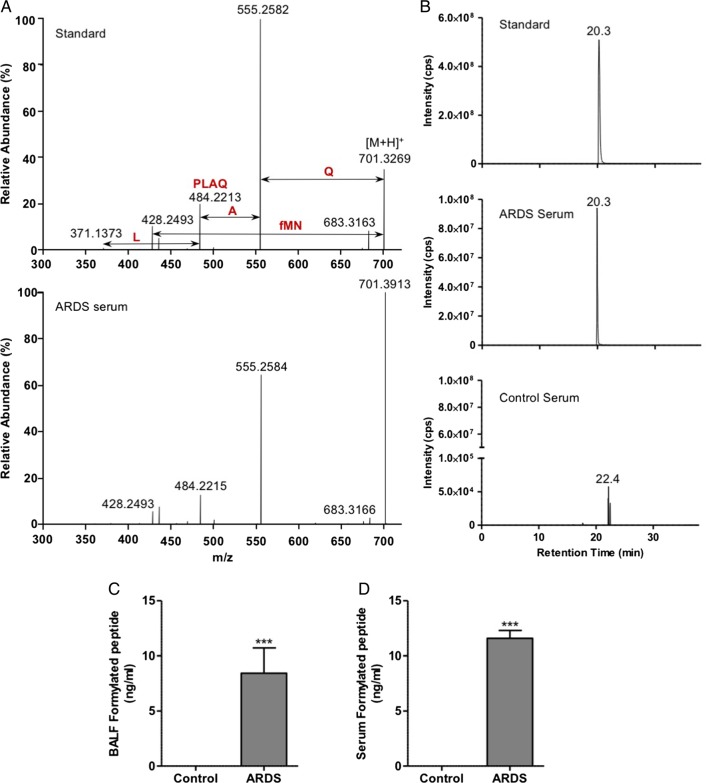
Elevation of formylated N-terminal peptides in patients with acute respiratory distress syndrome (ARDS). The N-terminal peptide fMNPLAQ (NADH2) was identified by liquid chromatography–tandem mass spectrometry (LC-MS/MS) on the basis of its fragmentation pattern (A) and chromatographic retention time (B) in comparison with a synthetic peptide standard. Quantitative analyses revealed significantly increased concentrations of the hexapeptide in bronchoalveolar lavage fluid (BALF) (C; n=10/group) and serum (D; control n=8 and ARDS n=7). Mann–Whitney test ***p<0.001.

Following the identification of free formylated N-terminal peptides in mitochondria, the work was extended to BALF and serum collected from patients with ARDS and appropriate healthy volunteer subjects as part of a proof of principle study (see online supplementary table S1). No significant difference in the age or sex of volunteer subjects compared with patients with ARDS was observed. The N-terminal hexapeptides from five of the mitochondrial proteins were putatively identified in patient samples (NADH subunits 2, 3 and 4, cytochrome b and ATP synthase subunit 8). In each case, the mass to charge ratio (m/z) and retention times of the peptides matched the synthetic standards; however, in the majority, MS/MS data were inconclusive. The NADH2 hexapeptide was the only one to be detected in BALF and serum from all samples from patients with ARDS. The identity of this mitochondrial peptide was confirmed by de novo sequencing by LC-MS/MS (figure 1A, B).

Quantification of the hexapeptide was undertaken by reference to a stable isotope-labelled internal standard (fMNP-[^13^C_6_, ^15^N]-LAQ). A calibration curve was constructed and found to be linear over the biological range with a regression coefficient of 0.997. LC-MS/MS analysis in plasma demonstrated a lower limit of detection of 0.02 ng/mL. There was a significant elevation in fMNPLAQ in BALF and serum of patients with ARDS, while in healthy volunteer subjects the hexapeptide was not detected (figure 1C, D). In view of the mixed patient population including four with positive bacterial culture of BALF fluid (methicillin-resistant *Staphylococcus aureus* (two patients), *Klebsiella pneumoniae* (one patient), *Enterobacter cloacae* (one patient)) comparison of formylated peptide burden between culture positive and negative patients was made. Importantly, concomitant bacterial infection did not influence fMNPLAQ concentration (see online supplementary figure S1A).

Given the spatial and biological associations between mitochondrial DNA (mtDNA) and formylated peptides both acting as proinflammatory DAMPs, mtDNA was also quantified in a smaller cohort, again as proof of principle. In keeping with the elevated levels of mitochondrial formylated peptides, mitochondrial DNA was increased in BALF and serum of patients with ARDS relative to healthy volunteers (see online supplementary figure S1B, C).

### Mitochondrial formylated peptides induce neutrophil chemotaxis in vitro and in vivo

**Figure 2:**
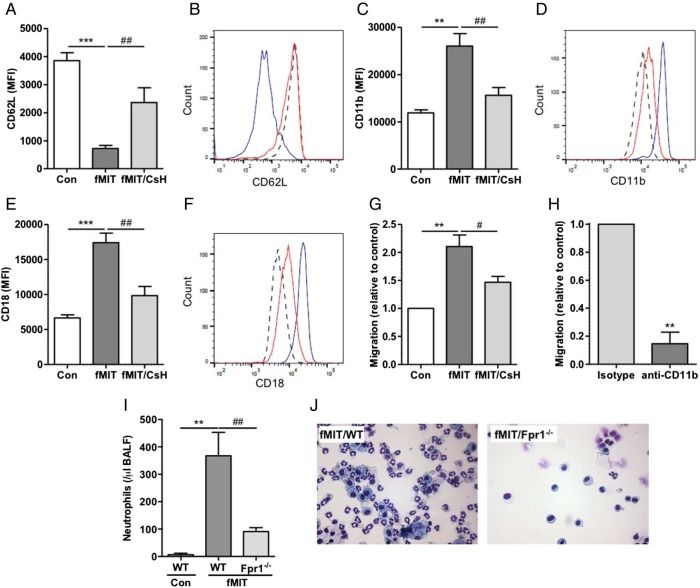
Mitochondrial formylated peptides alter adhesion molecule expression and chemotaxis in human neutrophils via a formyl peptide receptor 1 (FPR1)/Mac1-dependent mechanism and induce FPR1-dependent neutrophil recruitment in vivo. Mitochondrial formylated peptides (fMIT, 100 nM) induced CD62L shedding (A,B) and increased CD11b (C,D) and CD18 (E,F) expression as assessed by flow cytometry—effects that were inhibited by CsH (2.5 μM) (n=4 separate donors) (histograms—dotted line, control; blue line, fMIT; red line, fMIT/CsH). Neutrophil chemotaxis was increased in response to fMIT but inhibited by CsH (G) while anti-CD11b blocking antibody inhibited neutrophil migration towards isolated mitochondria (H), result expressed relative to transmigrated neutrophils treated with isotype control (n=3 separate donors). Administration of intratracheal fMIT (0.25 mg/kg) increased neutrophil numbers in bronchoalveolar lavage fluid (BALF) 12 hours after delivery in wild type (WT) mice relative to intratracheal vehicle control with alveolar neutrophils markedly reduced in fMIT-treated Fpr1−/− mice (I). Representative cytocentrifuge preparations shown, ×400 magnification (J) (n=5–10 per group). *Relative to vehicle control, #relative to fMIT/WT. #p<0.05, **/##p<0.01, ***p<0.001. (A,C,E and I) one way ANOVA with post hoc Newman Kuels test; (H) paired Student's t test.

Neutrophil chemotaxis is a multistep process in which CD62L (L-selectin) shedding is a prerequisite for rolling and firm adherence to vessel walls with increase in surface integrin expression facilitating subsequent transmigration. Assessment also serves as a well described assessment of neutrophil activation. Both synthetic mitochondrial formylated peptide (fMIT) and disrupted, isolated MTD induced human neutrophil CD62L downregulation and increased expression of the Mac-1 heterodimer CD11b and CD18 in vitro (figure 2A–F, see online supplementary figure S2A–C). Pharmacological inhibition with cyclosporin H (CsH), currently the most selective and potent FPR1 antagonist,^23^ ^24^ demonstrated that while not altering neutrophil phenotype in unstimulated cells (see online supplementary figure S3), CsH inhibited fMIT/MTD-mediated increase in CD11b and CD18 expression and CD62L downregulation (figure 2A–F, see online supplementary figure S2). Similarly, fMIT and MTD induced neutrophil chemotaxis—an effect abrogated by pretreatment with CsH (figure 2G, see online supplementary figure S2D). With regards to intracellular signalling, MTD induced phosphorylation of intracellular signalling proteins ERK, Akt and p38, effects blocked by CsH (see online supplementary figure S4A). Inhibition of ERK, p38 and PI3K but not Akt resulted in reduced neutrophil surface expression of components of CD11b and CD18 (see online supplementary figure S4B–D).

To determine whether mitochondrial formylated peptide induced migration was a Mac1-dependent process, neutrophils preincubated with either anti-CD11b antibody or appropriate isotype control were stimulated with MTD. Neutrophil migration was inhibited by the CD11b blocking antibody (figure 2H, p=0.0093) demonstrating that, in vitro, mitochondrial formylated peptide induced neutrophil chemotaxis is an FPR1/Mac1-dependent process.

To establish whether mitochondrial formylated peptides play a role in pulmonary neutrophil migration in vivo, fMIT was administered into the lungs of WT mice. Alveolar neutrophil numbers were significantly increased relative to vehicle control after 12 hours (figure 2I). Importantly, KC/CXCL1, the chemokine classically associated with pulmonary neutrophil recruitment, was undetectable in both groups (data not shown), indicating alternative signalling mechanisms for neutrophil migration. In Fpr1−/− mice the fMIT-induced alveolar neutrophil number was markedly reduced compared with WT animals (figure 2I, J).

### FPR1 is essential for neutrophil-dependent sterile lung injury in mice

To establish whether the FPR1-dependent alveolar neutrophil migration observed in response to mitochondrial formylated peptides was present in a disease context we utilised a model of hydrochloric acid induced lung injury. Acid-induced injury is regarded as a clinically relevant rodent model of acute lung injury mimicking the effects of acid aspiration of gastric contents. This model also allows determination of the role of mitochondrial formylated peptides in sterile inflammation and in an environment devoid of bacterial formylated peptides.^25–27^

**Figure 3:**
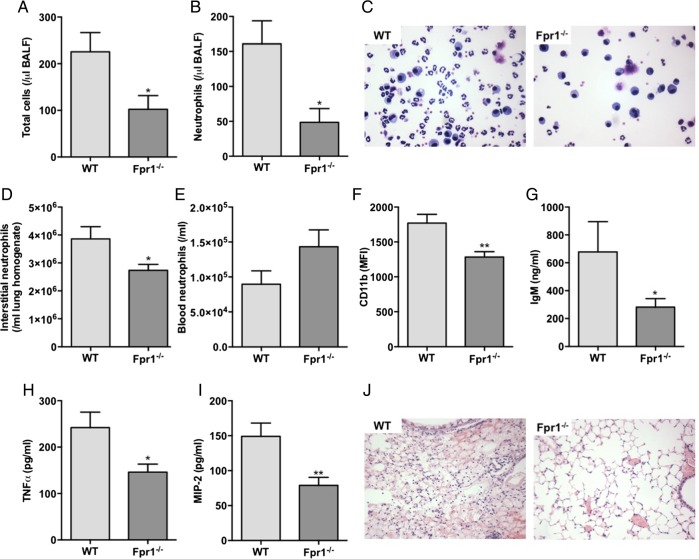
Sterile lung inflammation is attenuated in formyl peptide receptor 1 (Fpr1)−/− mice following intratracheal acid injury. Hydrochloric acid (pH 2.0) was instilled intratracheally with retrieval 24 hours after injury. Total cell count (A, p=0.042) and neutrophil number (B, p=0.020) in bronchoalveolar lavage fluid (BALF) (representative BALF cytocentrifuge preparations shown (×400 magnification) (C)) along with interstitial neutrophil numbers (D, p=0.047) were reduced in Fpr1−/− mice. No difference in circulating neutrophil number was observed (E, p=0.102). Interstitial neutrophil cell surface expression of CD11b was reduced in Fpr1−/− animals (F, p=0.007). BALF IgM (G, p=0.036), TNFα (H, p=0.030) and MIP-2 (I, p=0.0095) were quantified by ELISA and reduced in Fpr1−/− mice. Representative H&E sections (×100 magnification) (J). n=7–9/group, *p<0.05, **p<0.01, Student's t test.

Following instillation of intratracheal acid, there was evidence of host tissue damage and release of MTD with peak mtDNA levels observed 6 hours post injury (see online supplementary figure S5A). Alveolar neutrophil influx was greatest 24 hours after injury (see online supplementary figure S5B). Following acid-induced injury, Fpr1−/− mice had a marked reduction in total alveolar cell number and neutrophil numbers within the alveolar and interstitial lung compartments relative to WT animals (figure 3A–D). Importantly, no difference in circulating neutrophil numbers was observed (figure 3E). Similarly, no differences in alveolar, interstitial or circulating neutrophil numbers were seen between naïve Fpr1−/− and WT mice (see online supplementary figure S6). In keeping with the in vitro observation of Mac-1-dependent migration, Fpr1−/− mice displayed reduced CD11b expression on interstitial neutrophils (figure 3F).

Analysis of BALF demonstrated a reduction in pulmonary vascular leak with reduced soluble IgM levels in Fpr1−/− mice (figure 3G). The protective phenotype in these animals was further highlighted as the proinflammatory cytokines TNFα and MIP2 were reduced in the BALF of Fpr1−/− animals (figure 3H, I). Histological analysis of H&E-stained lung sections demonstrated reduced haemorrhage in Fpr1−/− mice (figure 3J).

**Figure 4:**
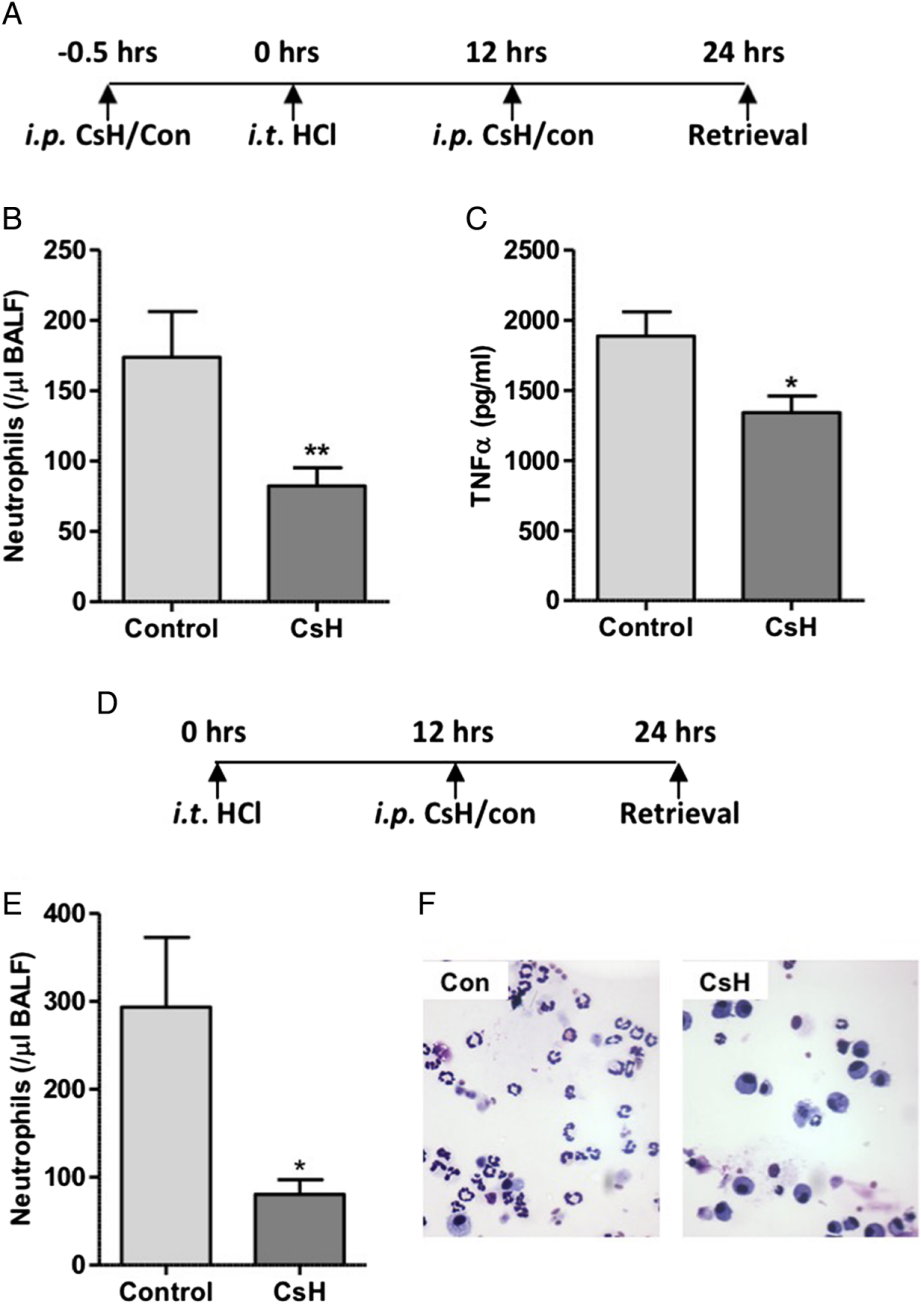
Delivery of formyl peptide receptor 1 (FPR1) antagonist cyclosporin H reduces neutrophil recruitment in acid-induced lung inflammation. Timeline of cyclosporin H (CsH) treatment during hydrochloric acid induced acute lung injury (A). CsH attenuated bronchoalveolar lavage fluid (BALF) neutrophil numbers (B, p=0.008) and TNFα levels (C, p=0.012) (n=12–16/group). In separate experiments CsH was administered intraperitoneally solely 12 hours after acid instillation with animals culled at 24 hours (D). BALF neutrophil number (E, p=0.022) was again reduced following CsH treatment. Representative cytocentrifuge preparations shown (×400 magnification) (F). n=7/group, *p<0.05, Student's t test.

Early phase neutrophil recruitment is characteristically associated with chemokine and cytokine mediated migration with later phase influx associated with pulmonary monocyte infiltration and activation of the stromal derived factor 1 (SDF1)/CXCR4 axis. Bone marrow recruited neutrophils, characteristic of late phase recruitment, often display a blunted chemotactic response to factors including the bacterial formylated peptide fMLF.^28–30^ To determine whether the reduction in alveolar neutrophil recruitment observed at 24 hours was solely as a result of inhibition of early phase recruitment, or whether FPR1 was implicated in the second phase of neutrophil recruitment, FPR1 antagonism was pharmacologically achieved with intraperitoneal CsH administered before and after the onset of lung injury. In keeping with results from acid injury to Fpr1−/− mice, pretreatment with CsH resulted in reduced alveolar neutrophil number and TNFα expression (figure 4A–C). To determine the effect of FPR1 antagonism on late phase neutrophil recruitment the antagonist was delivered 12 hours after acid injury (without pretreatment). Again, alveolar neutrophils were reduced relative to vehicle control treated mice (figure 4D–F). This reduction in neutrophil number (72.6% of control) is equivalent to that seen in mice pretreated with CsH (52.6%) and Fpr1−/− animals (69.8%), suggesting that the role of FPR1 in late phase recruitment is as important as it is in early phase migration when chemokines and MTD levels are at their peak (see online supplementary figure S5).

### FPR1 is present on pulmonary epithelium and contributes to lung injury

**Figure 5:**
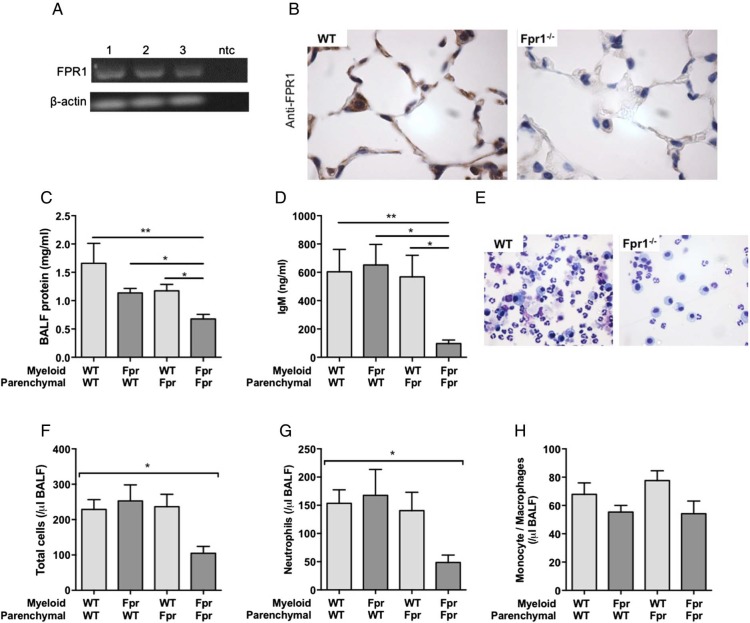
Formyl peptide receptor 1 (FPR1) is expressed on pulmonary epithelial cells with myeloid and parenchymal cell FPR1 contributing to neutrophil recruitment and alveolar protein leak during lung injury. Expression of epithelial FPR1 was determined by RT-PCR (A; 1–3=separate mice; ntc—no template control) of flow sorted mouse epithelial cells (EpCam1/E-cadherin positive) and immunohistochemistry (×1000 magnification) on mouse lung sections (B; representative images n=3 per group). Following re-engraftment bone marrow chimaeras (WT & Fpr1−/−) were injured with intratracheal HCl. Bronchoalveolar lavage fluid (BALF) total protein (C) and IgM (D) were quantified 24 hours after injury. Total BALF cell number (F) and neutrophil (G) and monocyte/macrophage count (H) were assessed (representative cytocentrifuge preparations of WT/WT and Fpr/Fpr BALF shown; ×400 magnification (E)). n=3–6/group *p<0.05, **p<0.001, one-way ANOVA.

Development of pulmonary oedema is multifaceted but key contributors include neutrophils, endothelial cells and alveolar epithelial cells. Depletion of neutrophils alone is sufficient to ameliorate protein leak in preclinical models of lung injury,^28^ ^31^ however there is increasing awareness of the role of parenchymal FPR1 expression and function in the context of acute colonic inflammation.^32^ We therefore characterised mouse lung epithelial FPR1 expression. Lungs of WT mice were collagenase digested, epithelial cells isolated and RNA extracted (see online supplementary figure S7) with FPR1 mRNA expression demonstrated by RT-PCR (figure 5A). Additionally, FPR1 protein was detected by immunohistochemistry in alveolar epithelial cells, as well as resident alveolar macrophages (figure 5B). Staining was absent in Fpr1−/− lung sections.

As alveolar epithelial and endothelial cell function plays an important contributory role in the development of pulmonary oedema in acute lung injury,^1^ we sought to determine the contribution of parenchymal FPR1 function in this process. Four-way Fpr1−/− and WT bone marrow chimeras were generated with acid-induced lung injury occurring 6 weeks after re-engraftment. Alveolar protein leak as assessed by total protein quantification and IgM within BALF demonstrated that myeloid and parenchymal FPR1 expression contribute to this process (figure 5C, D). Similarly, global loss of FPR1 appears necessary for reduction in neutrophil number. No difference in alveolar monocyte/macrophage number was observed between the groups (figure 5E–H).

## Discussion

Formylated peptides of bacterial and mitochondrial origin span the aetiological divides between sterile and infection-related diseases that contribute to ARDS. Delineation of their presence and the functional importance of their signalling through FPR1 in the pathogenesis of sterile lung inflammation is however required. In the context of pulmonary inflammation, Fpr1−/− mice are protected from cigarette smoking induced emphysematous changes, with associated reduction in neutrophil and macrophage number.^33^ Furthermore, a model of endotoxin-induced lung injury demonstrated a protective phenotype for Fpr1−/− mice.^34^ These studies propose an association with exogenous bacterial formylated peptides but no exploration of the effects of endogenous mitochondrial formylated peptides has been described in models of direct pulmonary injury.

Within the alveolar space, extracellular proteins are degraded by proteolytic enzymes both native to the environment and liberated by cell death during lung injury. While the presence of extracellular mitochondrial peptides acting as DAMPs has been surmised from the identification of free mitochondrial DNA,^14^ Fpr1−/− mouse studies,^15^ and in vitro observations on human neutrophils^35^ there has, to our knowledge, been no previous attempt to identify the functionally active formylated N-termini of these peptides within patient samples. Given the binding affinity of peptides to FPR1 is predominantly governed by the presence of a formylated methionine N-terminal amino acid determining this moiety's presence, and preservation in the context of extracellular proteolytic enzymes, it is important in delineating the peptides' biological activity.

Through LC-MS/MS analysis we identified the hexapeptide formylated N-terminus of NADH2, one of the 13 proteins encoded by the mitochondrial genome, and translated within the organelle. Furthermore, NADH2 was present in all patients with ARDS in both alveolar lavage fluid and the systemic circulation but undetectable in all healthy volunteer subjects in this proof of principle study. This therefore provides evidence that mitochondrial N-terminal formylated peptides are present in acute inflammatory disease and have the potential to interact with FPR1.

Considering the infective aetiology of some of the ARDS cohort, we were initially concerned that the formylated peptides detected in the clinical samples may arise from bacterial contamination rather than mitochondrial origin. BLAST searching showed some sequence similarities to bacterial proteins but this may be simply due to the high conservation of the peptide sequence motif. Further, the majority identified are hypothetical proteins while the BLAST search also gives no indication as to whether the protein has been subjected to post-translational modifications including formylation. Several independent factors reassured us that bacterial contamination contributing to formylated peptide detection was unlikely. First, no difference in formylated peptide levels was observed between sterile and culture-positive BALF samples. Second, cultures of healthy volunteer BALF samples frequently grew mixed oral commensals up to levels equivalent to those of the bacterial culture positive samples from patients with ARDS. Despite this, detectable formylated peptides were only present in the latter group. Third, the primers used for mtDNA PCR do not amplify bacterial DNA.^14^ Using these primers mtDNA was similarly elevated in the ARDS cohort. Fourth, the fMNPLAQ peptide was readily detected in the mitochondria isolated from sterile HepG2 cells. Taken together, we are satisfied that these separate observations support the conclusion that it is eukaryotic-derived mitochondrial peptides which are being detected and quantified. Further refinement of this methodological approach will be required to accurately determine global formylated peptide burden, which in turn should be applied to a larger mixed patient cohort to dissect detailed association with risk stratification or prognostic outcome. Larger sample size will also serve to address the limited number of patients included in this present study.

Although instinctively attributable to attenuation of neutrophil influx, the observed reduction in alveolar leak in Fpr1−/− mice may well be multifactorial. Neutrophil migration across endothelial and epithelial barriers with release of histotoxic mediators is alone sufficient to induce tissue oedema and pulmonary haemorrhage. Despite evidence of cellular crosstalk with innate inflammatory cells, the exact dynamics and key contributors to intrinsic alveolar epithelial cell dysfunction in the pathogenesis of ARDS remain poorly understood.^1^ The role of FPR1 in colonic epithelial homeostasis^32^ and formylated peptides in bronchial epithelial migration and contraction^36^ ^37^ and systemic vascular tone^38^ has recently been described. This highlights the influence of FPR1 function on epithelial function, suggesting its possible direct involvement in modulating alveolar epithelial permeability.

To determine the presence of FPR1 in mouse pulmonary epithelial cells, immunohistochemical staining of naïve mouse lung sections and RT-PCR of mRNA extracted from isolated lung epithelial cells were performed. Both demonstrated the presence of epithelial FPR1. To subsequently ascertain the relative contribution of myeloid and parenchymal FPR1 expression in the development of sterile acute lung injury bone marrow chimeras were made. Neutrophil influx and alveolar protein leak were reduced in only those mice with global FPR1 absence, demonstrating that myeloid and parenchymal cells contribute to lung inflammation. This observation is similar to recent work demonstrating that global MyD88 expression is required for neutrophil recruitment and bacterial clearance in a *Streptococcus pneumoniae* model of pulmonary infection through KC/CXCL1 and MIP2 dependent mechanisms.^39^

## Conclusion

Neutrophil migration is a highly orchestrated and regulated process dependent upon crosstalk between multiple cell types through a vast array of mediators and cell surface receptors. Viewed by the host as pathogenic mediators akin to those released by their bacterial ancestors, mitochondrial formylated peptides through pluripotent effects mediated by FPR1 may well be principal conductors in this inflammatory process. We have provided, for the first time, direct evidence of N-terminal formylated mitochondrial peptides in human disease with detection of peptides in the BALF and circulation of patient with ARDS. In concert with this, FPR1 is involved in neutrophil recruitment and alveolar leak in response to sterile injury. While mitochondrial formylated peptides induce direct, KC/CXCL1-independent, neutrophil migration in vivo, FPR1 mediates neutrophil-independent effects through parenchymal cells by mechanisms that are yet to be fully elucidated. Together, these data demonstrate that FPR1 warrants further examination and investigation as a potential therapeutic target in sterile lung inflammation.
