# Supplementary material for: Novel role for endogenous mitochondrial formylated peptide-driven formyl peptide receptor 1 signalling in acute respiratory distress syndrome
Source: Thorax. 2017 May 3;72(10):928–36. doi: 10.1136/thoraxjnl-2017-210030 (PMC5738532; doi:10.1136/thoraxjnl-2017-210030)
Supplement: supplementary data [file thoraxjnl-2017-210030supp001.pdf]

Online Data Supplement

Title

Novel role for endogenous mitochondrial formylated peptide-driven formyl peptide receptor 1 signalling in Acute Respiratory Distress Syndrome

Authors

David A. Dorward, Christopher D. Lucas, Mary K. Doherty, Gavin B. Chapman, Emma Scholefield, Andrew Conway Morris, Jennifer M. Felton, Tiina Kipari, Duncan Humphries, Calum T. Robb, A. John Simpson, Phillip D. Whitfield, Christopher Haslett, Kevin Dhaliwal, Adriano G. Rossi

MATERIALS AND METHODS

Clinical samples

Informed consent was received from participants, or next of kin in cases of incapacity, prior to inclusion in the study in accordance with the approved study protocol with the nature and possible consequences of the study explained. For collection of bronchoalveolar lavage fluid the bronchoscope was wedged in a subsegment corresponding to the area of radiological involvement and sterile saline (20ml) was instilled and the aspirate discarded, then 200ml of sterile saline was instilled in aliquots and the aspirate (representing an alveolar sample) retained. BALF was centrifuged at 700*g* for 10 min and the supernatant retained. Whole blood was collected into 3.8% sodium citrate and following centrifugation (350*g* for 20 min) serum was made by addition of 1M calcium chloride to the aspirated plasma layer. Samples were stored at -80°C prior to analysis. ARDS was defined at initial recruitment according to the American-European Consensus Conference definition and satisfy the current Berlin criteria with $PaO_2/FiO_2 \leq 300$mmHg, bilateral opacification seen on chest x-ray, positive end expiratory pressure (PEEP) $\geq$ 5cm H_2O and non-cardiogenic pulmonary oedema.[1,2]

Quantification of N-terminal formylated peptides

Biological and clinical samples (mitochondria (MTD), BALF and serum) were subjected to acetone precipitation. The acetone fraction, containing peptides, was dried under vacuum and reconstituted in 0.5% acetic acid. The peptides were then analysed by liquid chromatography-tandem mass spectrometry (LC-MS/MS) in positive ion mode using a Thermo LTQ-Orbitrap XL mass spectrometer coupled to a Waters nanoAcquity UPLC system with a linear gradient over 39 min (mobile phase A: 0.5% acetic acid in water; mobile phase B: 0.5% acetic acid in acetonitrile). N-formylated peptides were identified based on their accurate mass, retention times and characteristic fragmentation patterns compared to custom synthesised standards

(Peptide Protein Research Ltd, Fareham, UK). Quantification of the N-formylated peptide (fMNPLAQ, m/z = 701.3) was performed in selected ion monitoring (SIM) mode using an internal standard with a ^{13}C, ^{15}N universally labelled leucine residue (fMNP-[$^{13}C_6$, ^{15}N]-LAQ, m/z = 708.3). The concentration of the peptide was determined by comparison to a calibration curve run in parallel.

Quantification of mitochondrial DNA

DNA was extracted using Qiagen DNA Blood Minikit as per the manufacturer's instructions (blood and body fluids protocol, Qiagen, Manchester, UK). After thawing samples were spun for 10 min at 9500*g* and 200µl of serum or mouse BALF used (800µl human BALF sampled). DNA was eluted into a final volume of 40µl. Primers for human cytochrome C oxidase III (COXIII; MWG Eurofins, Vienna, Austria) were used as previously described[3] (Forward 5'-ATGACCCACCAATCACATGC, reverse 5'-ATCACATGGCTAGGCCGGAG) and analysed with 2X SYBRGreen in an ABI7900 Fast Real-Time PCR System (Applied Biosystems, Loughborough, UK) with a thermal of 1 cycle 95°C for 20 sec, 40 cycles of 95°C for 3 sec & 60°C for 30 sec with subsequent melt curve analysis. Mouse mtDNA was similarly quantified as described with primers for 16S rRNA (Forward 5′-CTAGAAACCCCGAAACCAAA, Reverse 5′-CCAGCTATCACCAAGCTCGT).[4] Absolute quantification of mtDNA was determined relative to a standard curve and expressed in copies per millilitre.[5]

Mitochondrial isolation

Mitochondria were isolated from HepG2 cells (ATCC #CCL-23) in accordance with manufacturer's instructions (ThermoScientific, Winsford, UK) and subsequently disrupted by three cycles of freeze thaw. Total protein was quantified by BCA protein assay

(ThermoScientific) with MTD used at a final concentration of 50μg/ml for *in vitro* neutrophil experiments.

Flow cytometry

Neutrophils were resuspended at 5 x 10^6/ml in PBS with divalent cations (Gibco, Loughborough, UK) and incubated with the FPR1 antagonist cyclosporin H (CsH) (2.5μM, Enzo Scientific, Exeter, UK), ERK inhibitors (PD0325901, PD184352), p38 inhibitor (SB203580), PI3K inhibitors (LY294002 and wortmannin) or Akt inhibitor (Akt-i); all 10μM (all Calbiochem, Watford, UK). Neutrophils were then stimulated with 100nM fMIT (fMMYALF; GenScript, Hong Kong), isolated mitochondria (MTD) or vehicle control for 30min (37°C). All samples were subsequently incubated on ice with anti-CD11b-AF488 (Clone ICRF44), anti-CD18-PE (TS1/18) and anti-CD62L-APC (DREG-56) (all BioLegend, London, UK) for 30 min prior to analysis by flow cytometry (BD FACS Canto, BD Biosciences, Oxford, UK).

Neutrophil chemotaxis

Neutrophil chemotaxis was conducted using a 96 well chemotaxis chamber fitted with a 3μm filter (Neuro Probe, Gaithersburg, MD, USA) as described.[6] Neutrophils, 3x10^6/ml (IMDM with 10% autologous serum) were pre-treated with CsH, anti-CD11b antibody (20μg/ml, ICRF44, BioLegend) or vehicle or istoype control for 30 min at 37°C and loaded in the upper well of the chamber. Chemoattractants MTD (50μg/ml), fMIT (100nM) or vehicle control were added in the lower well. Transmigrated neutrophils in the lower chamber were counted with a haemocytometer following 90 min incubation at 37°C 5% CO_2.

Intracellular calcium flux

Neutrophils, suspended in HBSS without divalent cations, were loaded with Fura-2/AM (2 µM; Invitrogen, Loughborough, UK) for 30 min, washed, and resuspended in HBSS with divalent cations at 2 x 10^6/ml. Intracellular calcium flux, in response to 100nM fMLF and PAF (both Sigma-Aldrich), was quantified using a spectrofluorimeter (Perkin Elmer, Waltham, USA), as described.[6] Cells were pre-treated with CsH (1µM) 2 min before addition of fMLF.

Western blotting

Neutrophils, $7x10^5$ cells per sample, were suspended in PBS with divalent cations and pre-treated with or without cyclosporin H (CsH, 2.5µM) for 30 min. MTD were added and cells incubated for 1 min at 37°C prior to cell lysis and protein extraction as described.[7] Briefly, following protein quantification by bicinchoninic acid assay samples were mixed with 4x NuPAGE sample buffer (ThermoScientific) and denatured at 96°C for 5 min. Gel electrophoresis in NuPAGE 4-12% Bis-Tris protein gels (Invitrogen) was performed and transferred to PVDF membrane prior to 1 h incubation with 5% dried milk in TBS-Tween (1%). Overnight incubation with primary antibody (pERK (9101S), pAkt (9271), p-p38 (9211), ERK (9102); Cell Signalling Technology, Boston, USA) at 4°C in 5% BSA in TBS-Tween was followed by incubation with secondary HRP-conjugated antibody (goat anti-Rabbit, Daco, Ely, UK). After washing membranes were visualised on photographic film (Kodak) using enhanced chemiluminescence (ECL Prime; Amersham, UK).

Mice

$Fpr1^{-/-}$ mice were a kind gift from Prof M. Perretti. $Fpr1^{-/-}$ mice were previously developed through insertion of a neomycin resistance cassette (1.1 kb) in reverse orientation inserted in

place of a 150bp open reading frame sequence within the first extracellular loop of the fourth transmembrane domain of the FPR1 receptor thereby inactivating FPR1 in all cell types.[8]

Tissue harvesting and processing

Mice were euthanased with an overdose of terminal anaesthesia followed by exsanguination by cardiac puncture. Harvested blood was mixed 1:1 with 3.8% sodium citrate prior to antibody staining for flow cytometry as described.[9] Lungs were lavaged with three boluses of 800µl sterile 0.9% NaCl and centrifuged at 850*g* for 5 min. Supernatants were stored at -80°C prior to further analysis. The cell pellets from all three washes were combined and resuspended in 500µl PBS without cations. Total cell count was determined by nucleocounter (Chemometec, Allerod, Denmark) and cytocentrifuge preparations were made, stained with DiffQuik and differential cell counts performed (300 cells per slide counted). To quantify interstitial neutrophil numbers the right ventricle was perfused with 5ml of sterile 0.9% NaCl prior to removal of the lungs. The lungs were subsequently placed in 500µl of 5mg/ml collagenase D (Roche, Burgess Hill, UK), sliced and incubated for 1 h at 37°C in a shaking heat block (400 rpm). Tissue was then disaggregated through a 19 gauge needle, centrifuged at 300*g* for 5 min and resuspended in 500µl ACK lysis buffer (Gibco). Following 5 min incubation on ice a further 500µl of PBS was added and cells centrifuged at 300*g* for 5 min. Cells were subsequently resuspended in 1ml of PBS and passed through a 40µm filter. The cell suspension (250µl) was incubated for 10 min with 2.5µl CD16/32 Fc Block (Clone 93, BioLegend) prior to antibody staining for flow cytometry.[9]

Neutrophil numbers in the vascular, interstitial and alveolar compartments were determined by flow cytometry. 20µl of whole blood, resuspended BALF cells, and cells from lung digest were stained with anti-CD45-PE (clone 30-F11), anti-CD11b-FITC (clone M1/70), anti-

Ly6G-Pacific Blue (clone 1A8) (all BioLegend) for 30 min prior to addition of 1ml FACSLyse for a further 30 min. Flow tubes were centrifuged at 300*g* for 5 min and pellets resuspended in 200µl PBS prior to analysis using BD LSRFortessa (BD Biosciences). To calculate absolute cell numbers 50µl Flow-Check fluorophores (Beckmann Coulter, Brea, USA) were added prior to running samples.

Analysis of BALF cytokines (KC/CXCL1, TNFα, MIP-2; R&D Systems, Abingdon, UK) and IgM (eBioscience, Hatfield, UK) were performed by enzyme linked immunosorbent assay (ELISA) as per manufacturers' instructions. Total protein was measured by bicinchoninic acid assay (Pierce, Northumberland, UK). For histological examination of mouse lungs organs were removed en bloc from the thoracic cavity and fixed overnight in 10% formalin (Sigma). Lungs were subsequently dehydrated by transfer into 70% ethanol prior to processing, paraffin fixation, sectioning and haematoxylin and eosin staining.

Assessment of epithelial FPR1 expression

RNA from flow-sorted epithelial cells was extracted using the Qiagen RNA extraction kit according to the manufacturer's instructions and quantified by nanodrop. Complementary DNA (cDNA) was made using the iScript cDNA Synthesis Kit (Bio-Rad) as per the manufacturer's instructions. PCR to detect FPR1 (Forward 5'-ATGTTCTAGGAGTCTACAAGATGG; reverse 5'-ATATATGAATTTGCACATGAACCA) and β-actin (Forward 5'-GCTTCTTTGCAGCTCCTTCGT; reverse 5'-GCGCAGCGATATCGTCATC) were conducted using primers and conditions previously described.[8] Reaction conditions were 1 cycle 94°C for 5 min; 30 cycles of 94°C for 30 s, 60°C for 30 s, and 70°C for 30 s and 1 cycle 72°C for 10 min. PCR products were run on a 2% agarose gel with a 100bp ladder. The

gel was run at 120V for 45 min until the leading edge reached the distal gel edge with products subsequently visualised using an ultraviolet illuminator.

For immunohistochemical staining to determine lung FPR1 protein expression in naïve WT and $Fpr1^{-/-}$ mice lungs were harvested en bloc and inflated with formalin prior to overnight fixation. Following paraffin embedding sections were deparaffinised in xylene prior to heat mediated antigen retrieval in NCL pH6 Epitope Retrieval buffer (Novocastra, Leica). Staining was performed in the Leica Bond Max automated immunostainer using the bond refine polymer detection kit and anti-FPR1 antibody (1:200; Orb213947, Biorbyt, Cambridge).

Bone marrow transfer

Non-autologous bone marrow transfer experiments were performed using 6 week old $Fpr1^{-/-}$ or WT female recipients with 8 week $Fpr1^{-/-}$ or WT male donors. Enrofloxacin (Baytril 2.5%, Bayer AG) was added to drinking water one week prior to irradiation and continued for the subsequent 5 weeks. All animals received a lethal dose of 1050 rad (10.5 Gy) delivered from a GammaCell 40E (MDS Nordian) with a cesium 137 source at a dose rate of 114 rad/min.[10] Two hours after irradiation $1x10^7$ donor bone marrow cells were injected intravenously in a volume of 200μl 0.9% NaCl. Six weeks after irradiation *i.t.* HCl (pH2.0) was instilled as outlined previously with subsequent tissue harvest after 24 h.

RESULTS

Table S1 – Demographic and clinical data for ARDS patients and health volunteer subjects

APACHE II Acute Physiology and Chronic Health Evaluation II, PEEP positive end expiratory pressure, PaO_2 partial pressure arterial oxygen, FiO_2 fraction of inspired oxygen; ICU, Intensive care unit. *Co-morbidities included coronary artery disease, congestive cardiac failure, chronic renal failure, chronic liver disease and diabetes; [A]t-test; [B]chi-squared;

	Health volunteers (n=10)	ARDS (n=12)	p value
Mean age (yrs) (range)	51.2 (22-67)	60.0 (27-78)	0.18[A]
Sex (M/F)	5/5 (50%)	6/6 (50%)	1.0[B]
Apache II score Mean (95% CI)		20 (18-22)	
PEEP (cm H_2O) Mean (95% CI)		9.9 (8.1-11.7)	
PaO_2/FiO_2 (mmHg) Mean (95% CI)		157 (120-193)	
Surgical diagnosis on admission (%)		50	
≥1 co-morbidity* (%)		75	
Mortality during ICU stay (%)		42	

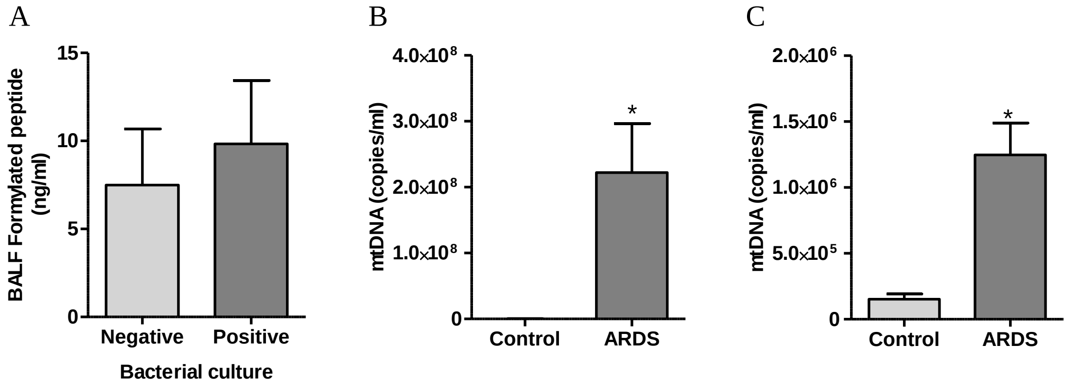

Figure S1. BALF formylated peptide levels are not influenced by concurrent bacterial infection whilst mtDNA is elevated in both BALF and serum in acute respiratory distress syndrome. No difference in detectable formylated peptide levels were observed between bacterial culture positive (n=4) and negative (n=6) BALF fluid in the ARDS cohort (**A**). Mitochondrial DNA in BALF, n=3 per group (**B**) and serum, n=6 per group (**C**) was quantified by qPCR in ARDS patients and healthy controls and was increased in the ARDS cohort in both compartments. Mann Whitney test, *p<0.05.

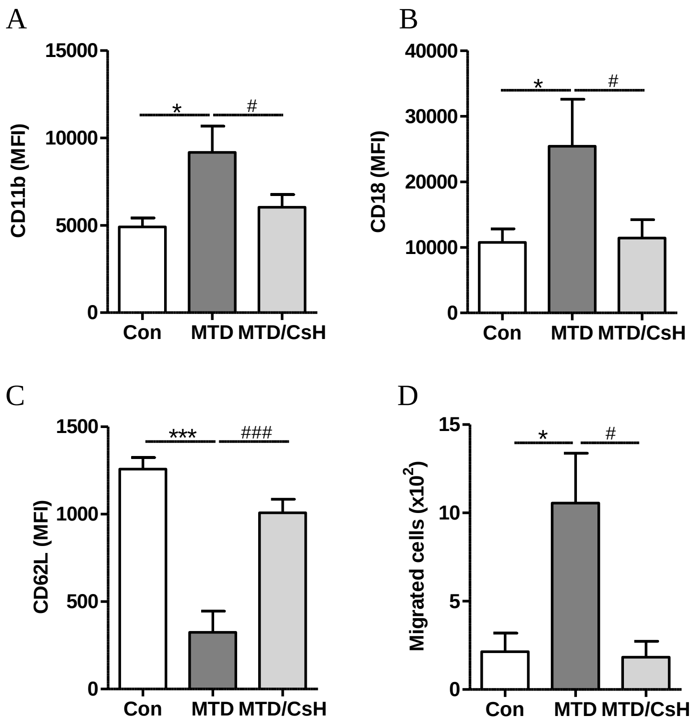

Figure S2. Isolated mitochondrial DAMPs induce neutrophil chemotaxis through FPR1-dependent mechanisms. Isolated human neutrophils were stimulated with mitochondrial DAMPs (MTD) resulting in upregulation of CD11b (**A**) and CD18 (**B**) expression along with loss of CD62L (**C**). These effects were inhibited by CsH (2.5µM). Similarly, MTD induced neutrophil chemotaxis, an effect inhibited by CsH (**D**). n=4 separate donors. $*/^{\#}$ $p<0.05$, $***/^{\#\#\#}p<0.001$. One way ANOVA with post hoc Newman-Keuls, *relative to unstimulated cells, #relative to MTD-stimulated cells.

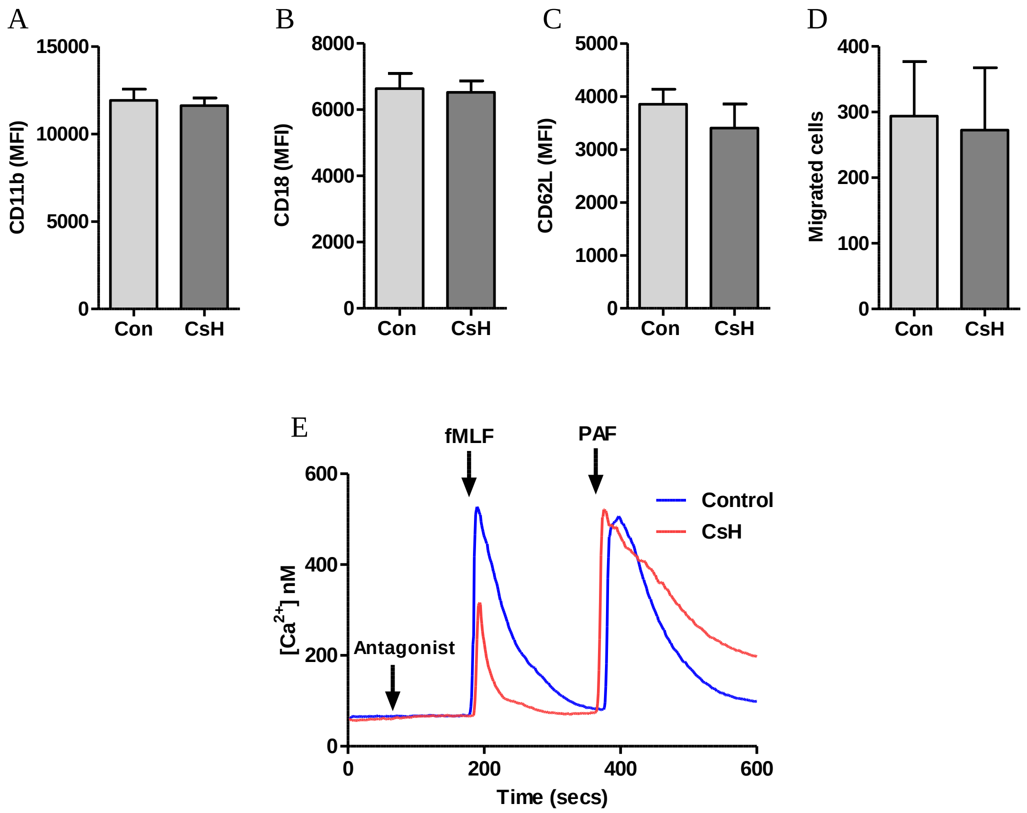

Figure S3. Cyclosporin H does not alter constitutive neutrophil function or interfere with response to alternative agonists. Expression of cell surface markers CD11b (**A**), CD18 (**B**) and CD62L (**C**) was assessed by flow cytometry on neutrophils incubated with CsH (2.5μM) or vehicle control with no difference in expression observed (n = 4 separate donors). Similarly, no difference in chemotaxis was seen between unstimulated neutrophils pre-incubated with CsH or vehicle control (**D**) (n=3 separate donors). Intracellular calcium flux was assessed in Fura-2AM-loaded neutrophils in response to fMLF and PAF (both 100nM) in cells pre-incubated for 2 min with CsH (1μM). No effect on PAF-induced intracellular calcium flux was observed whilst CsH inhibited fMLF-mediated response – individual traces shown of representative experiment (**E**).

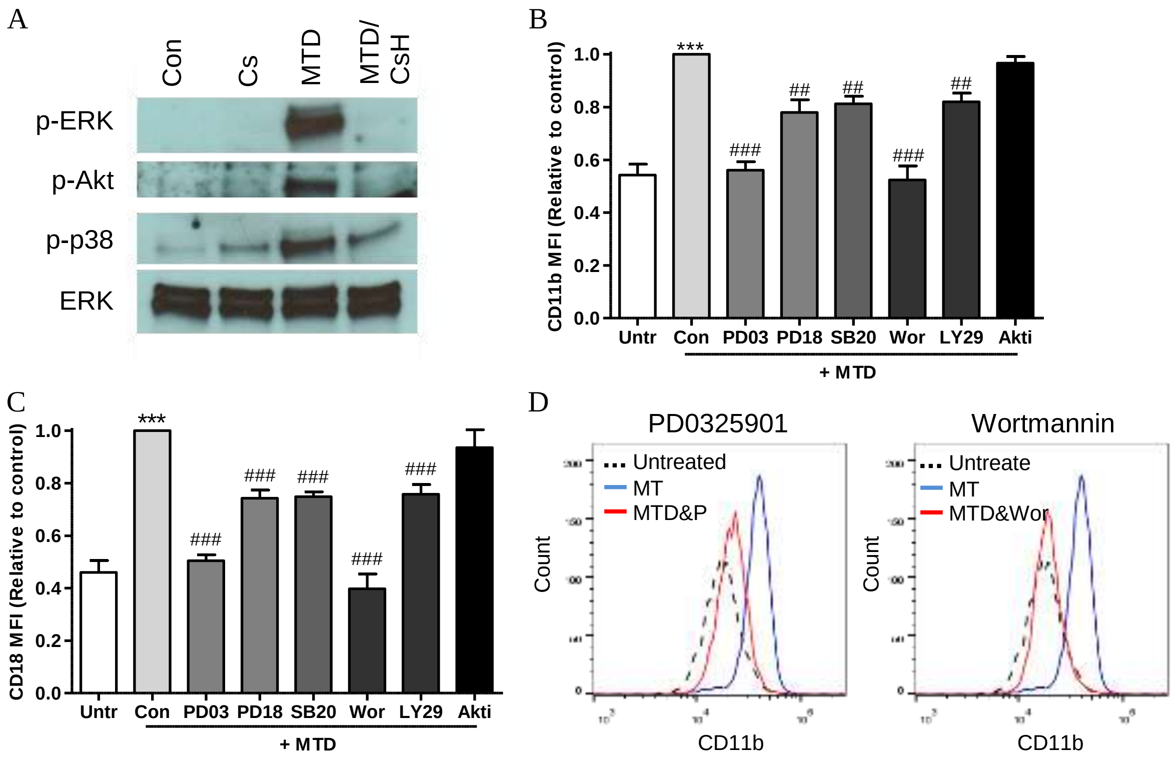

Figure S4. Mitochondrial formylated peptide-induced expression of β2-integrin heterodimer Mac1 is regulated by PI3K, MAPK and ERK signalling. MTD-induced phosphorylation of intracellular signalling molecules was examined by western blotting with neutrophil lysates harvested after 1 min incubation with MTD in the presence or absence of CsH (n=3 separate donors) (**A**). The effect of MTD on CD11b (**B**) and CD18 (**C**) expression when co-incubated with ERK inhibitors (PD0325901, PD184352), p38 inhibitor (SB203580), PI3K inhibitors (LY294002 and wortmannin) and Akt inhibitor (Akt-1) demonstrated a role for all but Akt in the regulation of CD11b and CD18 expression in response to MTD. Representative flow cytometry histograms (**D**). All compounds 10µM, n = 3 separate donors. $^{\#\#}p<0.01$, $^{***/\#\#\#}p<0.001$. One way ANOVA with post hoc Newman-Keuls, *relative to unstimulated cells, #relative to MTD stimulated cells.

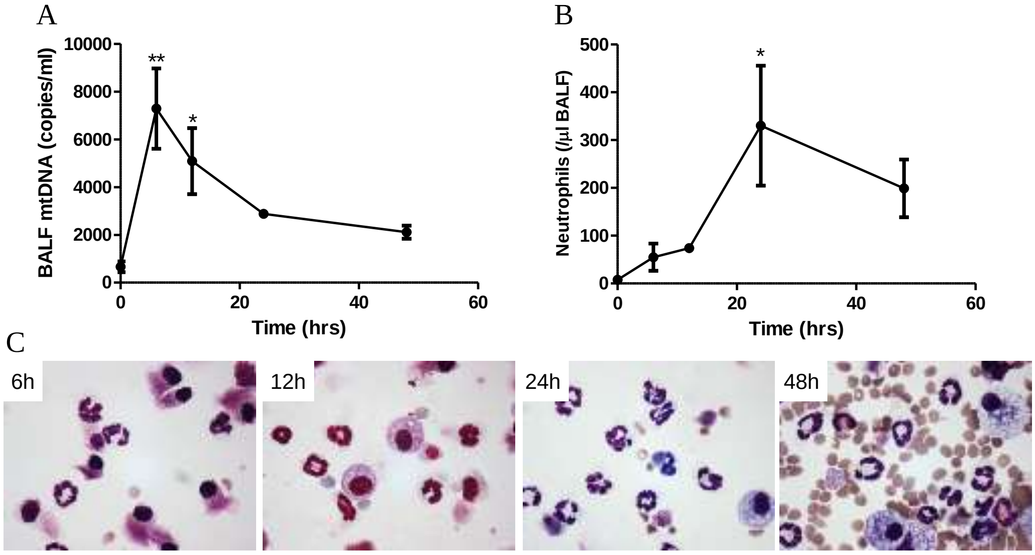

Figure S5. Time course of BALF mitochondrial DNA and neutrophil number following sterile acute lung injury with *i.t.* hydrochloric acid. Following *i.t.* HCl mice were culled after 6, 12, 24 and 48 h with BALF mitochondrial DNA quantified (**A**) and neutrophil numbers assessed (**B**). Representative cytocentrifuge preparations, 400x magnification (**C**). n=3 mice per time point. One way ANOVA with post hoc Dunnett's test. *p<0.05, **p<0.01.

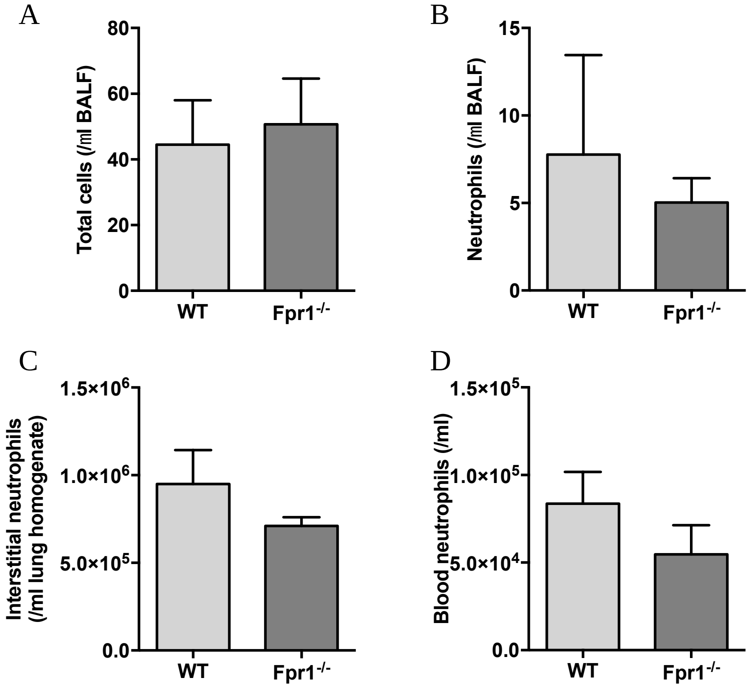

Figure S6. The Fpr1$^{-/-}$ phenotype does not alter neutrophil numbers within the lung or systemic circulation in naïve mice. No difference in total BALF cell count (**A**), BALF (**B**), interstitial (**C**) or circulating (**D**) neutrophils were observed in unstimulated, naïve mice (n=3 per group).

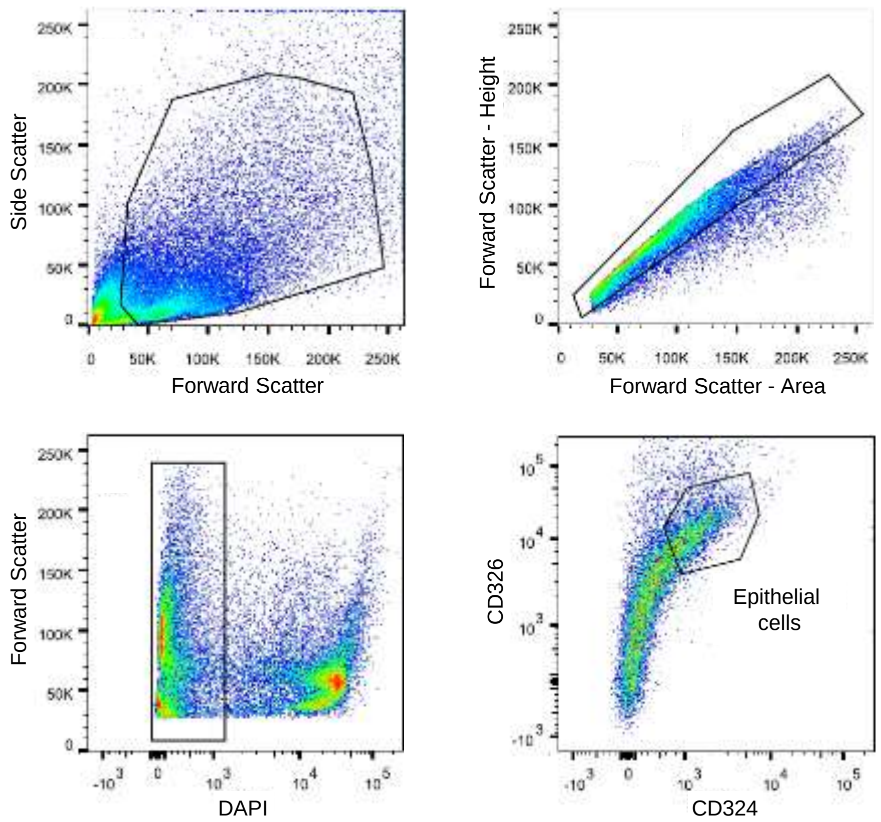

Figure S7. Gating strategy for the isolation of primary mouse lung epithelial cells. CD324 (E cadherin)$^{+}$ CD326 (EpCAM1)$^{+}$ epithelial cells were collected by flow sorting lung homogenates from naïve wild-type mice. Gating strategy for DAPI^{-}/CD326^{+}/CD324^{+} cells.

REFERENCES

1 Bernard GR, Artigas A, Brigham KL, *et al.* The American-European Consensus Conference on ARDS. Definitions, mechanisms, relevant outcomes, and clinical trial coordination. *Am J Respir Crit Care Med* 1994;149:818–24. doi:10.1164/ajrccm.149.3.7509706

2 ARDS Definition Task Force, Ranieri VM, Rubenfeld GD, *et al.* Acute respiratory distress syndrome: the Berlin Definition. *JAMA* 2012;307:2526–33. doi:10.1001/jama.2012.5669

3 Zhang Q, Raoof M, Chen Y, *et al.* Circulating mitochondrial DAMPs cause inflammatory responses to injury. *Nature* 2010;464:104–7. doi:10.1038/nature08780

4 Davidson BA, Vethanayagam RR, Grimm MJ, *et al.* NADPH Oxidase and Nrf2 Regulate Gastric Aspiration–Induced Inflammation and Acute Lung Injury. *J Immunol* 2013;190:1714–24. doi:10.4049/jimmunol.1202410

5 Chiu RWK, Chan LYS, Lam NYL, *et al.* Quantitative analysis of circulating mitochondrial DNA in plasma. *Clin Chem* 2003;49:719–26. doi:10.1373/49.5.719

6 Dorward DA, Lucas CD, Alessandri AL, *et al.* Technical advance: autofluorescence-based sorting: rapid and nonperturbing isolation of ultrapure neutrophils to determine cytokine production. *J Leukoc Biol* 2013;94:193–202. doi:10.1189/jlb.0113040

7 Leitch AE, Lucas CD, Marwick JA, *et al.* Cyclin-dependent kinases 7 and 9 specifically regulate neutrophil transcription and their inhibition drives apoptosis to promote resolution of inflammation. *Cell Death Differ* 2012;19:1950–61. doi:10.1038/cdd.2012.80

8 Gao JL, Lee EJ, Murphy PM. Impaired antibacterial host defense in mice lacking the N-formylpeptide receptor. *J Exp Med* 1999;189:657–62.

9 Lucas CD, Dorward DA, Tait MA, *et al.* Downregulation of Mcl-1 has anti-inflammatory pro-resolution effects and enhances bacterial clearance from the lung. *Mucosal Immunol* 2014;7:857–68. doi:10.1038/mi.2013.102

10 Kipari T, Hadoke PWF, Iqbal J, *et al.* 11β-hydroxysteroid dehydrogenase type 1 deficiency in bone marrow-derived cells reduces atherosclerosis. *FASEB J* 2013;27:1519–31. doi:10.1096/fj.12-219105
